# Improvement of Osteoporosis Screening among Inflammatory Bowel Disease Patients at Gastroenterology Fellows' Clinics

**DOI:** 10.1155/2020/7128932

**Published:** 2020-06-19

**Authors:** Antonios Wehbeh, Parkpoom Phatharacharukul, Nabil F. Fayad

**Affiliations:** ^1^Division of Gastroenterology and Hepatology, Indiana University School of Medicine, Indianapolis, IN, USA; ^2^Section of Gastroenterology and Hepatology, Richard L. Roudebush VA Medical Center, Indianapolis, IN, USA

## Abstract

**Introduction:**

Individuals with inflammatory bowel disease (IBD) have an increased risk of osteoporosis compared to the general population. We aimed to improve the osteoporosis screening rate in the IBD patient population of the gastroenterology (GI) fellows' continuity clinics.

**Methods:**

Baseline preintervention data were collected on patients seen from July through September of 2018. Four simplified criteria for osteoporosis screening were extrapolated from 3 national guidelines. Among patients who met any of these criteria, we determined the baseline screening rate. Fellows were then educated with a didactic session and handout material, and a standardized template was incorporated into clinic notes. Following this intervention, screening rates were reassessed from December 2018 through February 2019.

**Results:**

During the preintervention phase, fellows saw 80 patients with IBD. Dual-energy X-ray absorptiometry (DEXA) scan was obtained in 44% of IBD patients who qualify for screening at the county hospital clinic compared to 21% of veterans' clinic IBD patients. In the postintervention period, screening rates remarkably improved to 100% in the county hospital clinic and to 75% in the veterans' clinic. Overall, the screening rate increased by 56% (*P* < 0.001).

**Conclusions:**

A large percentage of IBD patients at risk for osteoporosis did not have appropriate bone mass density testing. Educating GI fellows and adding a template to clinic notes were effective in significantly improving the number of patients at risk of osteoporosis to receive appropriate screening test, a DEXA scan. Similar educational interventions should be considered for providers caring for IBD patients to prevent complications of osteoporosis in these patients.

## 1. Introduction

Inflammatory bowel disease (IBD) is frequently encountered in the practice of gastroenterologists. The incidence can vary depending on the population studied. For example, the annual incidence rates for Crohn's disease (CD) and ulcerative colitis (UC) in a Veterans Affairs (VA) population were 33 per 100,000 and 50 per 100,000 VA users, respectively [1]. The incidence in the general population of North America is reported to be up to 20.2 per 100,000 person-years for CD and 19.2 per 100,000 person-years for UC [2]. Patients with limited health literacy are more likely to have symptoms of active Crohn's, depressive symptoms and worse overall health [3]. In general, patients of the VA and county hospitals tend to have lower educational and socioeconomic status compared to the general population, and they could also have worse IBD-related outcomes [4].

Individuals with IBD have an increased risk of osteoporosis compared to the general population. The prevalence of osteoporosis in IBD patients is high, ranging from 13% to 42% [5–7]. The pathophysiology of decreased bone mineral density (BMD) in this patient population is not completely understood but thought to be multifactorial related to a state of chronic inflammation, corticosteroid use, nutritional deficiencies, and small bowel resections [8, 9].

Making a diagnosis of osteoporosis is important because it is a known risk factor for bone fractures, and patients with osteoporosis should receive appropriate treatment [10]. The presence of osteoporosis and osteopenia can be determined by measuring BMD, and this is commonly done using a DEXA scan. To exemplify the utility of BMD, if the BMD is one standard deviation lower than age adjusted mean, the relative risk of having a fracture increases by 1.6 to 2.6 [11]. Major gastroenterology societies, including the American Gastroenterology Association (AGA), the American College of Gastroenterology (ACG), and the European Crohn's and Colitis Organization (ECCO), have all recommended screening UC and CD patients for osteopenia/osteoporosis, although with small variations in defining the at-risk patients [5, 12, 13]. Given that the gastroenterologist is often regarded as the main provider for IBD patients, gastroenterologists and primary care physicians are equally responsible to recommend preventive measures and screening for osteoporosis, in addition to other healthcare maintenance issues [12]. However, studies are concerning for low osteoporosis screening rates and low adherence to the abovementioned guidelines [6, 11, 14, 15].

In this study, we aimed to measure the overall osteoporosis screening rate in the IBD patient population of the GI fellows' continuity clinics at our institution and implement measures to improve it.

## 2. Methods

This quality improvement study was developed and executed by the trainees in our Gastroenterology and Hepatology Fellowship Program within the context of our trainee continuity clinics at the affiliated county hospital and VA medical center. All trainees who participated in the initiative were gastroenterology fellows. As the project was designed as a quality improvement initiative, this study was exempt from the Institutional Review Board (IRB) review. This study was conducted in compliance with the ethical standards of our institution.

### 2.1. Inclusion/Exclusion Criteria

Baseline preintervention data were collected on patients seen over a 12-week period. We utilized the scheduling timesheets to determine the patients seen by the fellows in their continuity clinics at the two locations (VA clinic and county hospital clinic). We included all adult patients who had a confirmed diagnosis of IBD, as documented in GI clinic notes. The diagnosis of IBD is typically made based on well-known clinical, endoscopic, and histologic criteria. The electronic chart was reviewed and the following information was collected: age, sex, subtype of IBD (UC, CD, or indeterminate colitis), whether they met osteoporosis screening criteria, and whether a DEXA scan had been obtained in the prior 2 years (by any of the patient's providers). Patients who were less than 18 years of age and those who did not carry a diagnosis of IBD were excluded.

### 2.2. Determining Who Needs Osteoporosis Screening

To determine if a patient should have a DEXA scan, we referred to national osteoporosis screening guidelines. Because there are many risk factors and conditions that predispose to osteoporosis, we focused for the purposes of this study on the essential factors that apply for the majority of patients. We refer to those risk factors as the “simplified osteoporosis screening criteria.” Those simplified criteria were extrapolated from three guidelines (American College of Gastroenterology (ACG) 2017, United States Preventive Services Task Force (USPSTF) 2018, and National Osteoporosis Foundation (NOF) 2014). They include females aged 65 years or older, males aged 70 years or older, use of oral corticosteroid therapy in a dose ≥7.5 mg/day of prednisone equivalent for 3 or more consecutive months, and a previous bone fracture in adult life occurring spontaneously or from a trauma which normally would not result in a fracture in healthy individuals. The presence of any of these screening criteria was determined by retrospectively reviewing clinic notes, medical problem lists, radiology studies, and medications dispensed by pharmacy.

### 2.3. Quality Initiative Intervention

Among patients who met any of these criteria, we determined the baseline/preintervention osteoporosis screening rate, defined as the proportion of patients who had a bone DEXA scan in the past 2 years. Fellows were then educated by means of a didactic session along with handout material (Appendix A), and a standardized template was incorporated into their clinic notes (Appendix B). Following this educational intervention, screening rates were reassessed over another 12-week period by charts review. The postintervention screening rate was defined as the proportion of subjects who had DEXA scans performed within the past 2 years or ordered in clinic during the 12 weeks after the intervention. Each fellow reviewed another fellow's patients' encounters in both the preintervention and postintervention periods to ensure an unbiased data collection. The pre- and postintervention screening rates were compared, and *P* values were calculated using a two proportion *Z*-test in independent groups.

## 3. Results

### 3.1. Preintervention Baseline Characteristics

During the preintervention phase, fellows saw 743 patients, 80 of whom had IBD (36 had UC, 42 had CD, and 2 with indeterminate colitis). Of these 80 IBD patients, 45 were seen at the VA clinic and 35 were seen in the county clinic. Ninety-three percent at the VA clinic were male IBD patients compared to 37% at the county clinic. The VA patients were older than at the county clinic (mean age of 61.6 years versus 46.8).

### 3.2. Preintervention Screening Rates

A total of 45 IBD patients (56%) met at least one of the determined osteoporosis screening criteria, and 29 of those 45 were seen at the VA clinic. DEXA scan was obtained in 44% of IBD patients who qualify for screening at the county hospital clinic, compared to only 21% of veterans' clinic IBD patients. The most common indications for screening were age criteria and steroid use.

### 3.3. Postintervention Baseline Characteristics

In the postintervention period, 81 patients with IBD were seen (36 had UC, 42 had CD, and 3 with indeterminate colitis). More IBD patients were seen at the VA clinic (46 patients) compared to the county clinic (35 patients). The demographics were similar to those of the preintervention phase. Specifically, the majority (98%) of the VA clinic patients were male as compared to the majority being female (63%) at the county clinic. In addition, the VA patients were older than at the county clinic (mean age of 62.7 years versus 45.1).

### 3.4. Postintervention Screening Rates

Forty-seven IBD patients (58%) met at least one of the determined osteoporosis screening criteria. Screening rates remarkably improved to 100% in the county hospital clinic (relative risk (RR) = 2.28, *P* < 0.001) and to 75% in the veterans' clinic (RR = 3.62, *P* < 0.001). Overall, the combined screening rate in both clinics increased from 29% to 85% (RR = 2.91, *P* < 0.001) (Table 1).

## 4. Discussion

Our study showed that a large proportion of IBD patients seen by GI fellows met one or more criteria for osteoporosis screening, consistent with other studies in the literature that also report high proportion among IBD patients [6].

However, before implementation of the educational intervention, the majority of patients did not receive an appropriate DEXA scan, indicating low compliance rate with existing ACG and other guidelines. Previous studies have also been published on low osteoporosis screening rates in this patient population. In a nationwide study on IBD patients in the Veterans Health Administration, only 15% received a DEXA scan [14]. Similarly, in a large Swiss cohort, osteoporosis screening rate varied among six medical centers, from 11% to 62% [11]. We theorize that the low compliance rate in our group is most likely from not having sufficient time to address all health maintenance issues during follow-up visits, in which complex medical decision-making is often required to manage patients' gastrointestinal illness. In addition to screening for osteoporosis, this health maintenance includes appropriate vaccinations, screening for depression and anxiety, and melanoma screening. However, the low compliance rate could also be due to lack of knowledge of osteoporosis screening recommendations or due to deferring DEXA testing to primary care physicians. Of note, the DEXA testing rate in the county clinic was higher, possibly because of a higher proportion of female patients compared to the veterans' clinic.

### 4.1. Study Strengths and Limitations

Postintervention, osteoporosis screening rate improvement was impressive, increasing overall by 56%. The main strength of this quality improvement project is that the intervention was simple to implement, yet very effective in increasing the rate of DEXA scans from 29% to 85%. This potentially could lead to the ability to identify more patients with osteoporosis and refer them for appropriate treatment to prevent bone fractures. A single educational session, using a PowerPoint presentation and giving handout material, served to educate and remind the fellows about existing guidelines and reinforce the importance of osteoporosis screening. It was easy to incorporate a brief template into the clinic notes via the two electronic medical record (EMR) systems at the two clinic locations. This template prompted the GI fellows to review charts and inquire from patients about risk factors for low BMD and if appropriate, order a DEXA scan. Our data is in line with other reports where similar interventions (consisting of provider education, chart reminders, EMR-based prompts, and patient education) had a positive impact on BMD screening rates, which in one study resulted in rate increase from 10.8 to 81.1% [16–18].

We are aware of some limitations of this study. First, some patients may have received medical care and DEXA scans at other hospitals in the community, and this would not be captured in our chart review. However, we expect this number to be very small as most veterans get all their care at the VA hospital, and similarly, most patients of the county clinic receive their care at the county hospital. Second, we collected the data retrospectively which could lead to the underestimation of patients who met the screening criteria as the data regarding steroid use or previous fractures might be missing. Finally, our study design did not allow us to determine the longevity of the positive effect of this intervention, before we would need to reimplement the intervention.

## 5. Conclusions

In conclusion, it was evident that many of the GI fellows' IBD patients at risk for osteoporosis were not receiving appropriate BMD screening. We were able to remarkably improve the rate of obtaining screening DEXA scans from 29% to 85% through a simple intervention of educating GI fellows and adding a template to the EMR clinic notes. Our hope is that these positive results would lead to an early diagnosis and treatment of osteoporosis, before ensuing complications and morbidities. This quality initiative could potentially be replicated successfully in other settings, such as clinics of GI attendings or advanced practice providers, both at our institution and at other institutions, and has a larger-scale preventative impact to improve outcomes of IBD patients.

## Figures and Tables

**Table 1:** Demographic data, characteristics, and DEXA screening rate for IBD patients who are at risk of osteoporosis.

	Preintervention phase (12 weeks)			Postintervention phase (12 weeks)		
	VA	County	All	VA	County	All
Total clinic patients	368	375	743	402	403	805
No. of IBD patients	45	35	80	46	35	81
UC	21	15	36	23	13	36
Crohn's	22	20	42	20	22	42
Indeterminate	2	0	2	3	0	3
Male, *N* (%)	42 (93.3%)	13 (37.1%)	55 (68.8%)	45 (97.8%)	13 (37.1%)	58 (71.6%)
Age (years), mean ± SD	61.6 ± 15.0	46.8 ± 14.4	55.1 ± 16.5	62.7 ± 16.4	45.1 ± 12.7	55.1 ± 17.2
No. of IBD patients who met DEXA screening criteria, *N* (%)	29 (64.4%)	16 (45.7%)	45 (56.3%)	28 (60.9%)	19 (54.3%)	47 (58.0%)
Steroid criteria	13	14	27	11	18	29
Age criteria	19	3	22	19	1	20
Fracture criteria	0	1	1	0	2	2
Patients who received appropriate screening DEXA scan, *N* (%)	6 (20.7%)	7 (43.8%)	13 (28.9%)	21 (75.0%)	19 (100.0%)	40 (85.1%)

IBD, inflammatory bowel disease; UC, ulcerative colitis; SD, standard deviation; DEXA scan, dual-energy X-ray absorptiometry

## Data Availability

The data used to support the findings of this study are available from the corresponding author upon request.

## Supplementary Materials

Supplementary Materials Appendix A: sample material from the didactic presentations and handouts that were used for educating the fellows. Appendix B: standardized clinic template that was incorporated into the fellows' electronic clinic notes, as part of the quality initiative intervention.
