# Supplementary material for: Improvement of Osteoporosis Screening among Inflammatory Bowel Disease Patients at Gastroenterology Fellows' Clinics
Source: Adv Prev Med. 2020 Jun 19;2020:7128932. doi: 10.1155/2020/7128932 (PMC7321501; doi:10.1155/2020/7128932)
Supplement: Supplementary Materials — Appendix A: sample material from the didactic presentations and handouts that were used for educating the fellows. Appendix B: standardized clinic template that was incorporated into the fellows' electronic clinic notes, as part of the quality initiative intervention. [file 7128932.f1.zip › 7128932.f1/Appendix A.pdf]

Intro

- Patients with IBD often consider their gastroenterologist to be their primary care provider.

- Therefore, it is becoming increasingly important that preventive care and healthcare maintenance issues be co-managed by both the gastroenterologist and primary care team.

-This includes appropriate vaccinations, screening for depression and anxiety, melanoma screening, and screening for osteoporosis- the main emphasis of our quality improvement project.

Some risk factors for osteoporosis: full list more extensive…

2364 Osteoporos Int (2014) 25:2359–2

Table 1 Conditions, diseases, and medications that cause or contribute to osteoporosis and fractures

Lifestyle factors		
Alcohol abuse	Excessive thinness	Excess vitamin A
Frequent falling	High salt intake	Immobilization
Inadequate physical activity	Low calcium intake	Smoking (active or passive)
Vitamin D insufficiency		
Genetic diseases		
Cystic fibrosis	Ehlers-Danlos	Gaucher's disease
Glycogen storage diseases	Hemochromatosis	Homocystinuria
Hypophosphatasia	Marfan syndrome	Menkes steely hair syndrome
Osteogenesis imperfecta	Parental history of hip fracture	Porphyria
Riley-Day syndrome		
Hypogonadal states		
Androgen insensitivity	Anorexia nervosa	Athletic amenorrhea
Hyperprolactinemia	Panhypopituitarism	Premature menopause (<40 years)
Turner's and Klinefelter's syndromes		
Endocrine disorders		
Central obesity	Cushing's syndrome	Diabetes mellitus (types 1 and 2)
Hyperparathyroidism	Thyrotoxicosis	
Gastrointestinal disorders		
Celiac disease	Gastric bypass	Gastrointestinal surgery
Inflammatory bowel disease	Malabsorption	Pancreatic disease
Primary biliary cirrhosis		

ACG 2017 Clinical Guideline: Preventive Care in Inflammatory Bowel Disease

- Patients who have IBD should be screened based on established guidelines for the general population. Osteoporosis screening guidelines differ between societies, however, DEXA testing is recommended by the National Osteoporosis Foundation for postmenopausal women aged 65 and men aged 70 and older

- BMD measurement (with a DEXA scan) is recommended … specifically in those who have used oral corticosteroid therapy for longer than 3 consecutive months in a dose ≥7.5 mg/day of prednisone-equivalent in the absence of baseline BMD measurement

Am J Gastroenterol. 2017 Feb;112(2):241-258.

Indications for osteoporosis screening in our project based on combination of 3 guidelines

1. Female age 65 years old and older
2. Men age 70 years old and older
3. Patients who have used oral corticosteroid therapy for longer than 3 consecutive months in a dose >/= 7.5mg/day of prednisone-equivalent in the absence of baseline BMD measurements
4. Previous bone fracture in adult life occurring spontaneously, or a fracture arising from trauma which, in a healthy individual, would not have resulted in a fracture) including vertebral and hip fractures
