# Supplementary material for: Improvement of Osteoporosis Screening among Inflammatory Bowel Disease Patients at Gastroenterology Fellows' Clinics
Source: Adv Prev Med. 2020 Jun 19;2020:7128932. doi: 10.1155/2020/7128932 (PMC7321501; doi:10.1155/2020/7128932)
Supplement: Supplementary Materials — Appendix A: sample material from the didactic presentations and handouts that were used for educating the fellows. Appendix B: standardized clinic template that was incorporated into the fellows' electronic clinic notes, as part of the quality initiative intervention. [file 7128932.f1.zip › 7128932.f1/Appendix B.pdf]

Clinic Template

1. Does the patient have IBD? YES or NO

If patient has IBD complete the next questions, otherwise delete the text below.

2. If IBD, is it Crohn's or is it UC? ---

3. Does the patient meet criteria for DEXA scan ?
   - Age criteria? YES or NO
   - Steroids? YES or NO
   - Fracture? YES or NO

4. Is there a DEXA scan done in past 2 years? YES or NO

5. If meets criteria and no DEXA in system, please order test if appropriate
